# Differences in gut microbiota profile between women with active lifestyle and sedentary women

**DOI:** 10.1371/journal.pone.0171352

**Published:** 2017-02-10

**Authors:** Carlo Bressa, María Bailén-Andrino, Jennifer Pérez-Santiago, Rocío González-Soltero, Margarita Pérez, Maria Gregoria Montalvo-Lominchar, Jose Luis Maté-Muñoz, Raúl Domínguez, Diego Moreno, Mar Larrosa

**Affiliations:** 1 Department of Basic Sciences, European University, Madrid, Spain; 2 Research Group on Nutrition, Physical Activity and Health, School of Doctorate Studies and Research, European University, Madrid, Spain; 3 Department of Physical Activity and Sports Science, Alfonso X El Sabio University, Villanueva de la Cañada, Spain; Harvard Medical School, UNITED STATES

## Abstract

Physical exercise is a tool to prevent and treat some of the chronic diseases affecting the world’s population. A mechanism through which exercise could exert beneficial effects in the body is by provoking alterations to the gut microbiota, an environmental factor that in recent years has been associated with numerous chronic diseases. Here we show that physical exercise performed by women to at least the degree recommended by the World Health Organization can modify the composition of gut microbiota. Using high-throughput sequencing of the 16s rRNA gene, eleven genera were found to be significantly different between active and sedentary women. Quantitative PCR analysis revealed higher abundance of health-promoting bacterial species in active women, including *Faecalibacterium prausnitzii*, *Roseburia hominis* and *Akkermansia muciniphila*. Moreover, body fat percentage, muscular mass and physical activity significantly correlated with several bacterial populations. In summary, we provide the first demonstration of interdependence between some bacterial genera and sedentary behavior parameters, and show that not only does the dose and type of exercise influence the composition of gut microbiota, but also the breaking of sedentary behavior.

## Introduction

Sedentary lifestyle is associated with a high incidence of chronic diseases such as cardiovascular disease, cancer and diabetes. Physical exercise is a powerful preventative and treatment intervention that is known to be effective in generating metabolic and immune health benefits [1]. The gut microbiota is essential for processing dietary components and has a major role in shaping the immune system [2]. Accordingly, the role of gut microbiota in determining host health and development of disease has been gaining interest in the last decade; however, whether gut microbiota is a target for the therapeutic benefits of exercise remains unknown [3].

Dysbiosis or imbalance in gut microbiota has been associated with many diseases, among which are ulcerative colitis, Crohn's disease, colon cancer [4,5], metabolic syndrome [6], type I and type II diabetes [7,8], cardiovascular disease [9], allergy, asthma, eczema and autism [10]. While it remains unclear in many cases whether the imbalance in gut microbiota is a cause or a consequence of the disease, interventions using probiotics and/or prebiotics are able to ameliorate symptoms of some diseases [11], indicating that manipulation of gut microbiota could be a viable therapeutic strategy [12]. Accordingly, there is a concerted effort to determine the composition of the core healthy gut microbiota and the factors that modify it [13,14]. Along this line, a very recent study that analyzed the gut microbiota of 1135 individuals by deep sequencing found the influence of gut microbiota on 31 intrinsic factors, 12 diseases, 19 drug groups, 4 smoking categories and 60 dietary factors [14]. Although a total of 207 factors were evaluated in this work, physical exercise as a factor that may influence gut microbiota composition was not evaluated. Several studies in experimental models have addressed the relationship between gut microbiota composition and physical exercise. In 2007, Bäckhed et al. suggested the existence of a microbiota-muscle axis that protects mice from obesity [15]. Consistent with this notion, the bacterial abundance of *Clostridiaceae* and *Bacteroidaeae* families and *Ruminoccocus* genus were found to be negatively associated with blood lactate levels in exercised animals, whereas a positive association was found for *Oscillospira* genus [16]. Evans et al. observed that changes in the *Firmicutes*/*Bacteroidetes* ratio exerted by exercise were inversely proportional to the distance traveled by animals [17]. Furthermore, voluntary wheel running was found to induce changes in the abundance of 2150 taxa and was able to offset the decrease in gut microbial richness in mice induced by exposure to polychlorinated biphenyls [18], and also increased *Lactobacillus* genus and *Blautia coccoides-Eubacterium rectale* group in rats [19]. Moreover, it seems that the changes exerted by physical exercise depend on the physiological state of the individual. Accordingly, forced exercise increased microbiota richness in obese, hypertensive and normal rats, which was dependent on their physiological state [16]. Additionally, alterations to the microbiota exerted by exercise in rats on high-fat diet were different to those produced in rats on normal diet [20], and those produced in diabetic mice were different to those produced in their control counterparts [21]. Collectively, these findings indicate that modulation of the microbiota by exercise depends not only on the physiological state of the individual, but also on the diet. Finally, it has been observed that exercise induces more effective changes in the microbiota in juvenile rats than in adult rats [22]. The only human study carried out to date that addresses alterations in microbiota by physical exercise compared the gut microbiota of healthy male controls with that of professional rugby players [21]. A greater diversity of microbiota species was found in rugby players, which would indicate a greater state of health in athletes since loss of diversity has been associated with several diseases [23,24], while increased diversity is associated with a better state of health in older people [25]. Nevertheless, in the human study [21], the large difference between diets of athletes and healthy controls (differences in energy, carbohydrates [both complex and simple], protein, fat and fiber) makes it difficult to isolate the effects of exercise against other divergent factors.

The main objective of the present work was to perform an observational study comparing the composition of the gut microbiota between individuals who did not practice any physical exercise and those who at the very least practiced exercise at the minimun dose recommended by the World Health Organization (WHO) [26]. Because hormonal status and sex are factors that influence gut microbiota, the study was conducted in premenopausal women.

## Materials and methods

### Subject characteristics

Forty premenopausal women were recruited according to inclusion criteria: age 18–40 years and body mass index (BMI) 20–25 kg/m^2^. Criteria for physical exercise were as follows: sedentary women who did not perform the minimal exercise established as healthy by WHO, ie 3 days of exercise per week for 30 minutes at a moderate intensity [26]; and for active women, those who performed at least 3 hours of physical exercise per week. Exclusion criteria were any kind of pathology, previous gastrointestinal surgery, antibiotics intake during three months prior to the study, smoking, prebiotics, probiotics, vegetarian or vegan, nutritional or ergogenic complements, pregnancy or lactation. All subjects were Caucasian. The Ethics Committee for Clinical Research of the Ramón y Cajal Hospital (Madrid, Spain) approved the study. Written and informed consent was obtained from all participants. The study is registered in Clinicaltrials.gov with the accession number NCT02901912.

### Physical activity and sedentary time determination

Levels of physical activity performed by both groups of women were measured with an ActiSleep V.3.4.2 accelerometer. (Actigraph, Manufacturing Technology Inc., Shalimar, FL). This allowed us to verify in an objective manner the presorting of sedentary and active individuals as it determines body movements in all three axes. Acceleration, energy expenditure, intensity of physical activity and body position was recorded. Results were analyzed with Actilife software (Actilife6, Actigraph). Recordings were performed for 7 days (5 weekdays and 2 weekend days). Participants were instructed to wear the accelerometer on the right wrist all day except when they had a shower or performed pool activities. Data were considered valid when they contained a record of at least five valid days including at least one weekend day, and at least 10 hours of activity were recorded. The time-sampling interval (epoch) was every minute during the 7 days. According to previous reports, a cut-off of <100 counts/minute was chosen to categorize sedentary time, which typically includes activities such as sitting or working quietly (eg, reading or typing on a computer) [27]. A sedentary bout was defined as 1 or more consecutive minutes with less than 100 counts/minute. A sedentary break was considered when there was an interruption in sedentary time of at least 1 minute with values above 100 counts/minute. Physical activity was considered as light when the activity count was 100–1951 counts/minute, and moderate-to-vigorous when counts were 1952–5724 counts/minute according to the methodology of Freedson et al. [28].

### Food frequency questionnaire

Dietary pattern characterization of the participants was carried out using a food frequency questionnaire (FFQ) with 97 food items. The FFQ was given to participants to complete the day they donated the fecal sample. Data from the FFQ were analyzed using Dietsource software 3.0 (Novartis, Barcelona, Spain) to obtain the total energy ingested (in kcals) of proteins, fat, carbohydrates, fiber and ethanol intake.

### Body composition measurement

Body composition was evaluated by dual-energy X ray absorptiometry (DEXA) (GE Healthcare, Madison, WI). Participants were provided with a gown and were placed in the middle of the table in a supine position. The measures of body composition assessed in the study were as follows: total body fat mass, estimated visceral adipose tissue (VAT), total muscle mass as well as fat and muscle mass distribution in the trunk and extremities. The following indices were calculated using the obtained values: adiposity index (AI) = total fat/height^2^; muscular mass index (MMI) = total muscle mass/height^2^ and appendicular muscular mass index (AppMMI) = muscle mass in arms+legs/height^2^.

### Stool collection, DNA extraction and sequencing

Participants were provided with a plastic device to collect stool samples, which were stored at -80°C until extraction. DNA was extracted using the commercial Power Soil DNA Kit (Mobio, CA) with a bead-beating homogenizer (Bullet Blender® Storm, Next Advance, NY). The concentration and purity of DNA was measured using the Quant-iT PicoGreen dsDNA Assay Kit (ThermoFisher Scientific, Waltham, MA).

### Sequencing and bioinformatics

Microbiota composition analysis of fecal samples was performed by amplifying the hypervariable regions V3 and V4 of the 16s rRNA gene using the primer pair 5’-TCGTCGGCAGCGTCAGATGTGTATAAGAGACAG-3' and 5'-GTCTCGTGGGCTCGGAGATGTGTATAAGAGACAG-3’ [29] to generate amplicons of 459 bp that were sequenced on a MiSeq Illumina platform (Illumina, San Diego, CA). Sequence outputs were analyzed using the Quantitative Insights into Microbial Ecology (QIIME) program, version 1.9.1 [30], using QIIME default parameters except for split library demultiplexing (phred quality threshold of 20 and better). The 16s pair-end reads were assembled using the script multiple_join_paired_ends.py, which joins forward and reverse demultiplexed reads. The output file was processed for quality filtering by split_libraries_fastq.py. High quality sequences were grouped into Operational Taxonomic Units (OTUs) with a sequence identity threshold of 97%, and taxonomy was assigned by interrogating the high quality sequences with the Greengenes database (13_5). Beta-diversity was evaluated by calculating weighted and unweighted Unifrac distances [31]. To study alpha-diversity, Shannon and Simpson diversity indices and Chao1 rarefaction estimators were calculated. Raw data in fastq format are available with the accession number PRJNA350839in the NCBI Biosample database (https://www.ncbi.nlm.nih.gov/bioproject/350839).

### Enterotype classification

Participants were classified into “enterotypes” according to the relative abundance of genera and Partitioning Around Medoids (PAM) using the Jensen-Shannon distance based on described methodology [32]. The results were assessed for the optimal number of clusters using the R package NbClust [33] and the Calinski-Harabasz index [34].

### Quantitative PCR analysis

Quantitative PCR (qPCR) analysis was carried out on a CFX Connect™ Real-Time PCR Detection System (BioRad, Barcelona, Spain) using SYBR Green I chemistry (BioRad) in 20 µL reactions containing one microliter (1–10 ng) of DNA template and 200 nM of primers. Cycling parameters were 95 °C for 10 minutes, followed by 40 cycles of 95°C for 15 seconds, 1 minute at the established annealing temperature and 72°C for 45 seconds. Subsequently, melting curve analysis was performed, in which fluorescence was measured as the temperature increased from 50°C to 95°C. For bacterial quantification, standard curves were made using serial dilutions of a known DNA concentration corresponding to a known number of bacteria for each group or bacterial species. The calculation of the percentage for each bacterial species or group was performed considering the quantity of bacteria obtained per gram of stool obtained with a universal primer pair as 100% [35]. For *Bifidobacterium longum* and *Roseburia hominis* detection, primers were manually designed considering the conserved regions in the 16s rRNA gene and the hypervariable regions to avoid cross-reaction with phylogenetically close species. To do this, the nucleotide sequences in FASTA format (.fas) were obtained from the NCBI nucleotide database. Primer sets were tested in silico with Testprime 1.0 against the small subunit ribosomal RNA SILVA database [29] and with MFE-primer-2.0 report [36]. Neither primer pair set matched to other bacterial species, while *Bifidobacterium longum* primers matched to the *longum* and *infantis* subspecies. The efficiency of PCR amplification for both primer pairs was calculated from the slope and intercept of a standard curve prepared using serial dilutions of DNA extracted from *Bifidobacterium longum* (DMS 20219) and *Roseburia hominis* (DMS 16839) acquired from the Leibniz Institute DSMZ-German Collection of Microorganism and Cell Culture. Primer sequences, product size, annealing temperatures and efficiencies are listed in S1 Table. Melting curves obtained from DNA and fecal samples are shown in S1 Fig.

### Fecal water content

To obtain the fecal water content, 500 mg of each fecal sample was weighed and then lyophilized in a freeze-drier (Christ Alpha 1–2 LD, Osterode am Harz, Germany). Water content was expressed as the percentage of weight loss of stool samples.

### Enzymatic fecal activity

Fecal enzymatic activity was determined using the API-ZYM system (Biomerieux, Marcy l'Etoile, France) according to the method described by Delgado et al. for fecal samples [37] and the manufacturer´s instructions. The API-ZYM system is a semi-quantitative method that determines 19 enzyme activities: alkaline phosphatase, acid phosphatase, phosphohydrolase, esterase, lipase, lipase-esterase, leucine arylamidase, valine arylamidase, cystine arylamidase, trypsin, α-chymotrypsin, α-galactosidase, β-galactosidase, β-glucuronidase, α-glucosidase, β-glucosidase, N-acetyl-β-glucosaminidase, α-mannosidase and α-fucosidase.

### Statistical analysis

Statistical analysis was carried out using QIIME version 1.9.1, SPSS software 20.0 (SPSS, Chicago, IL) and the R statistical package 3.3.1. Variable normal distribution was assessed using the Shapiro-Wilk test. Comparisons of variables between sedentary and active subjects were analyzed by Student's t-test (two-tailed). Those variables that did not meet normality were analyzed using the Mann-Whitney test. Bivariate correlation analyses were assessed using the Spearman correlation coefficient (Spearman Rho) using R, employing diet, body composition physical activity parameters and those genera that were present at least in the 80% of the samples. Linear regression analysis was performed for those microbial taxa that were differentially significant between sedentary and active subjects. The stepwise backward elimination method was employed using the following covariates: diet variables (total energy, protein, carbohydrates, lipids, fiber, ethanol, vegetables, cereals, dairy products, fruits, processed meat, beer and coffee), fecal water, body composition parameters (BMI, percentage of body fat, AI, VAT, MMI and AppMMI) and physical activity parameters (moderate physical activity, vigorous physical activity, average hourly kilocalories expended, total time in sedentary bouts, average time per sedentary bout, maximum time per sedentary bout, total time in sedentary breaks, average time per sedentary break, number of sedentary breaks, maximum time per sedentary break and total energy expenditure). Multiple regression models were built when multiple variables were predictive. Principal Component Analyses (PCoA) of community structure (β-diversity) using the unweighted and weighted distance metric were generated by QIIME [30], visualized by EMPeror [30] and analyzed by permutational multivariate analysis of variance (PERMANOVA) using the script compare_categories.py. Significance was set initially at p<0.05. In multivariate analyses (diet, microbial taxa [family and genus], correlation analysis and fecal enzymatic activities), p values were corrected with the Benjamini-Hochberg false discovery rate.

## Results

### Participant’s characteristics

Forty women were enrolled in the present study: 19 active (ACT) and 21 sedentary (SED). Anthropometric characteristics were similar between groups and no significant differences in age (ACT = 30.7±5.9 years vs SED = 32.2±8.7 years), weight (ACT = 63.45±11.2 kg vs SED = 61.4±9.0 kg), height (ACT = 165.6±8.65 cm vs SED = 163.7±6.3 cm) and body mass index (BMI) (ACT = 24.4±4.5 vs SED = 22.9±3.0) were found between groups.

### Body composition analysis

Analysis of body composition parameters indicated significant differences in almost all adiposity and muscle parameters between sedentary and active women (Table 1).

**Table 1:** Fat and muscle body composition parameters.

	ACT	SED	p
Sample size	n = 19	n = 21	
BFP (%)	27.4±4.8	34.5±4.7	0.001
BFM (kg)	16.1±5.9	20.79±5.5	0.027
VAT (g)	206.9±118.9	330.7±141.0	0.011
AI (kg/m^2^)	6.12±1.7	7.7±2.0	0.018
MMI (kg/m^2^)	15.3±1.7	13.3±1.2	0.001
AppMMI (kg/m^2^)	6.7±1.1	5.6±0.6	0.001

BFP: body fat percentage; BFM: body fat mass; VAT: estimated visceral fat; AI: adiposity index; MMI: muscular mass index; AppMMI: appendicular muscular mass index. Values are means±standard deviation. ACT = active group: SED: sedentary group.

### Dietary habits

Data on annual food consumption were collected with a FFQ and analyzed using Dietsource 3.0. Diets of both sedentary and active women conformed to the parameters of a balanced diet (Table 2), where the appropriate percentage of macronutrients is considered as follows: 10–15% proteins, 50–60% carbohydrates and 30–35% lipids [38]. No significant differences in macronutrient consumption and total energy were found between groups; however, the quantity of fiber ingested by the active group was significantly higher than that of the sedentary group (p = 0.005) (Table 2), while no differences were found for ethanol intake. Regarding the results by food groups, there was a significantly greater consumption of fruits (p = 0.027) and vegetables (p = 0.037) by the active group, while the sedentary group ingested significantly greater amounts of processed meats (p = 0.007) (Table 2).

**Table 2:** Consumption of macronutrients, fiber, ethanol and main food groups.

	Active	Sedentary	p value
Sample size	n = 19	n = 21	
Total Energy consumption (kcal)	2093±549	2277±647	0.358
Carbohydrates (%)	47.4±6.5	44.9±5.0	0.190
Proteins (%)	17.3±1.6	16.2±1.6	0.195
Lipids (%)	35.4±6.3	38.8±5.35	0.083
Fiber (g)	30.9±11.0	21.4±7.1	0.005
Ethanol (g)	4.31±4.7	2.9±3.7	0.519
Fruits (s/d)	3.72±2.19	2.29±1.45	0.027
Vegetables (s/d)	4.29±2.09	3.08±1.22	0.037
Legumes (s/d)	0.13±0.12	0.23±0.16	0.063
Cereals (s/d)	2.09±0.96	1.90±1.24	0.609
Nuts (s/w)	1.90±1.96	1.45±1.78	0.463
Dairy products (s/d)	2.71±1.67	2.04±1.23	0.170
White meat (s/w)	2.60±1.35	2.78±1.43	0.713
Red meat (s/w)	0.23±0.10	0.30±0.21	0.205
Processed meat (s/w)	4.99±1.95	7.23±2.70	0.007
Fish (s/w)	5.97±2.36	6.35±3.44	0.695
Shellfish (s/w)	1.19±0.50	1.19±0.39	0.995
Eggs (s/w)	2.74±1.39	2.82±1.39	0.854
Pastries (s/w)	4.89±4.7	6.84±3.07	0.147

s/d: servings per day; s/w servings per week. Data represent the means±standard deviation.

### Physical activity

When physical activity was compared between groups, there was a significant difference in the percentage of moderate physical activity in the active group (29.96±5.61%) versus the sedentary group (22.82±3.64%) (p = 0.039), while there were no significant differences in light physical activity measurements. The average expenditure was higher in the active group (145±31 kcal/h) than in the sedentary group (105±22 kcal/h), and consequently the total energy expenditure was also higher for active women (3432±911 kcals) than for sedentary women (2523±982 kcals) (p = 0.048). No significant differences were found for the remaining determined variables. Differences in sedentary behavior were also reflected in the variables “maximum time per sedentary break on working days” (SED = 691±96 min vs ACT = 515±91 min; p = 0.005), “average time per sedentary break on working days” (SED = 81.15±14.71 min vs ACT = 58.82±15.89 min; p = 0.038), and in the variable “total time in sedentary bouts on working days” (SED = 311±21 min vs ACT = 367±35 min; p = 0.027).

### Microbiota metagenomic analysis

**Fig 1:**
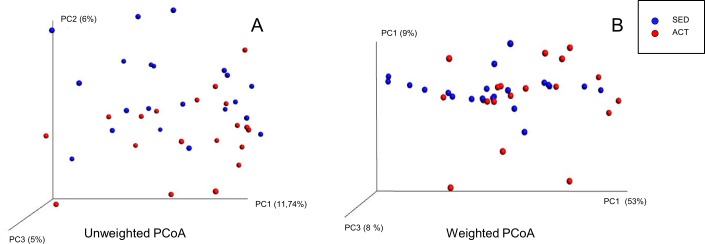
**Principal Coordinates Analysis (PcoA) plots of unweighted (A) and weighted (B) Unifrac distance metrics obtained from sequencing the 16s rRNA gene in fecal samples**. Axes represent percentage of data explained by each coordinate dimension.

**Fig 2:**
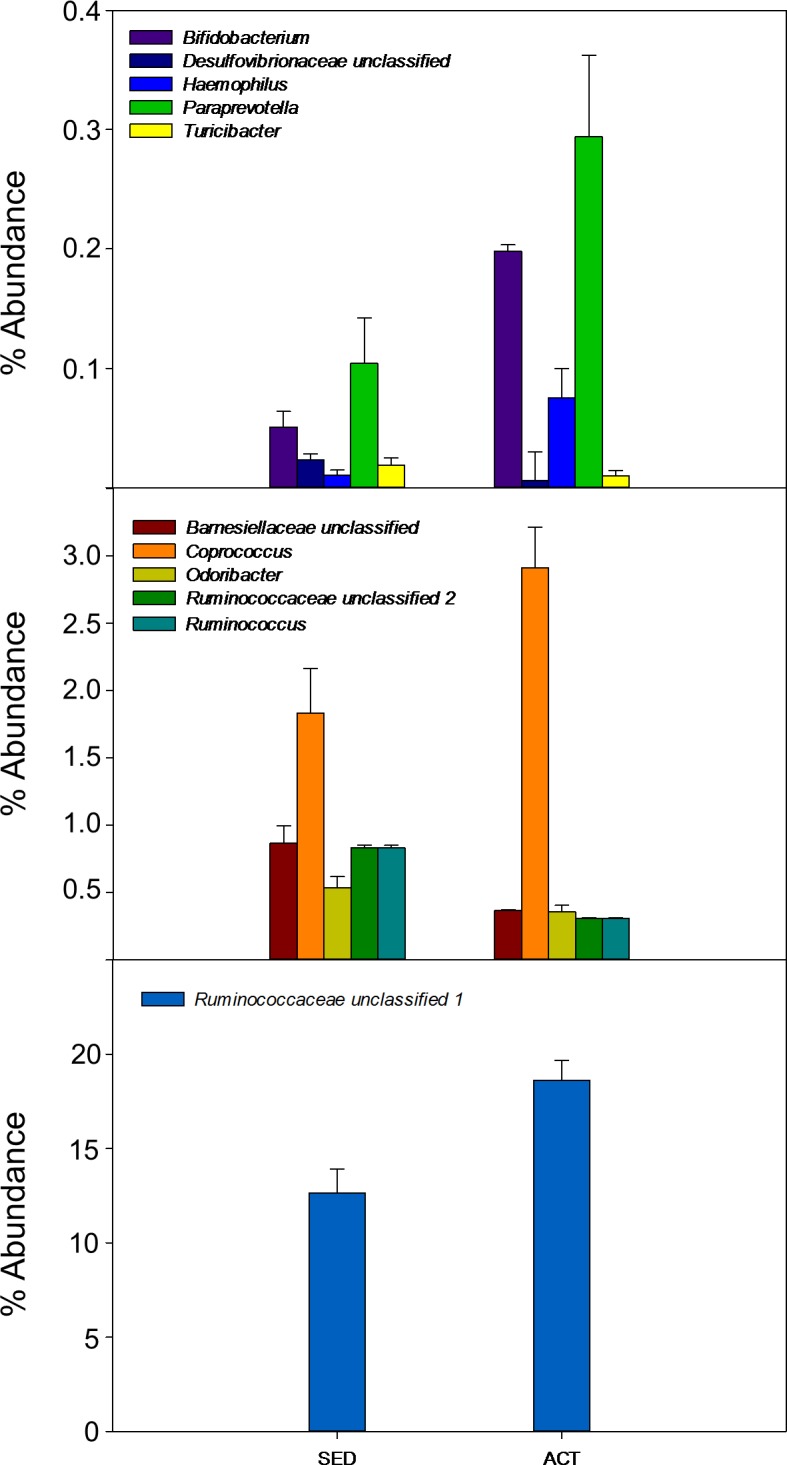
Abundance of different bacterial genera in fecal microbiota of active and sedentary women. Only statistical significant genera with an adjusted value p<0.001 are represented.

**Fig 3:**
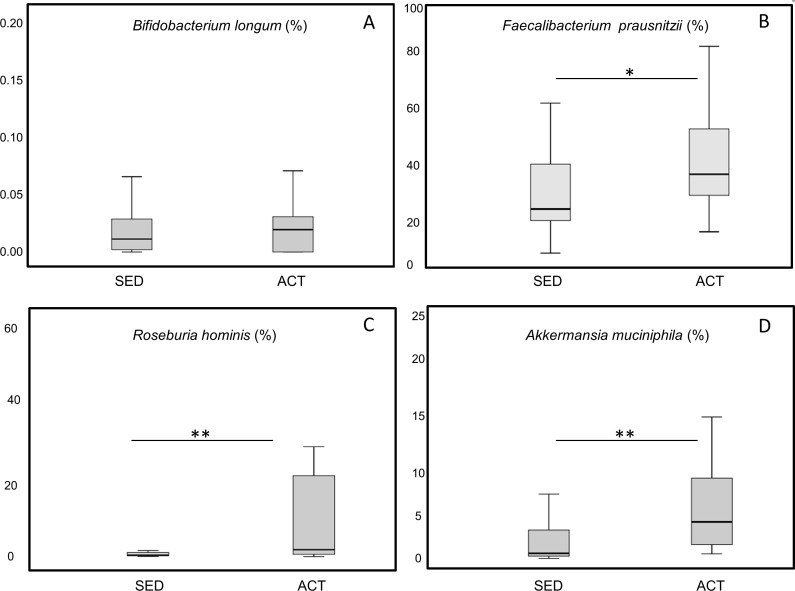
Changes induced in relative abundance of gut bacteria species by lifestyle in women. (A) *Bifidobacterium longum*, *(*B) *Faecalibacterium prausnitzii*, (C) *Roseburia hominis*, (D) *Akkermansia muciniphila*. Data were log transformed and analyzed by t-test * p<0.05 **p<0.01.

The average number of reads per sample was 26,890. Rarefaction curves based on observed species, Chao1, and phylogenetic distance whole tree measures were virtually saturated, suggesting sufficient sequencing depth (S2 Fig). No significant differences between the active and sedentary groups were detected in alpha diversity parameters (Observed, Chao1) or Shannon diversity index, indicating that species richness was similar between both groups (S2 Fig). Regarding β-diversity, no significant differences were detected in the Principal Coordinates Analysis (PCoA) based on unweighted Unifrac distance metrics (Fig 1A) (p = 0.141, pseudo-F = 1.15) or on weighted Unifrac distance metrics (Fig 1B) (p = 0.052, pseudo-F = 2.61). A total of 15 phyla were detected, in order of presence: *Bacteroidetes* (54%), *Firmicutes* (44%), *Proteobacteria* (0.96%), *Tenericutes* (0.39%), *Verrucomicrobia* (0.11%), *Euryarchaeota* (0.08%), *Actinobacteria* (0.07%), *Lentisphaerae* (0.06%), *Cyanobacteria* (0.050%), *Spirochaetes* (0.04%), *Fusobacteria* (0.014%), *Elusimicrobia* (0.009%), *Synergistetes* (0.007%), *kTM7* (0.003%), and *Acidobacteria* (0.0001%). *Acidobacteria* (2 subjects), *Elusimicrobia* (2 subjects) and *Spirochaetes* (2 subjects) phyla were detected only in sedentary subjects. At the phylum level, no significant differences were observed between the two groups, although there was a trend towards a higher presence of *Firmicutes* (p = 0.085) and a lower presence of *Bacteroidetes* (p = 0.076) in active women (Table 3). Likewise, the relative abundance of *Firmicutes*:*Bacteroidetes* ratio did not significantly differ between groups (p = 0.115). Significant differences were found in two bacterial families, *Barnesiellaceae* (p = 0.001) and *Odoribacteraceae* (p = 0.009), with a higher presence in sedentary women (Table 3). At the genus level, there were significant differences in eleven genera: *Bifidobacterium*, *Barnesiellaceae*, *Odoribacter*, *Paraprevotella*, *Turicibacter*, *Clostridiales*, *Coprococcus*, *Ruminococcus*, and two unknown genera of *Ruminococcaceae* family (Fig 2). Given the importance of some bacterial species in health, the presence of *Bifidobacterium longum* (Fig 3A), *Faecalibacterium prausnitzii* (Fig 3B), *Roseburia hominis* (Fig 3C), *Akkermansia muciniphila* (Fig 3D) was measured by qPCR. Analyses revealed a more significant abundance of *F*. *prautznnii* (p = 0.029) (Fig 3B), *R*. *hominis* (p = 0.005) (Fig 3C) and *A*. *muciniphila* (p = 0.002) (Fig 3D) in active than in sedentary women.

**Table 3:** Bacterial phyla and families in feces of active and sedentary women.

TAXA	ACT (%)	SED (%)	p value
*BACTEROIDETES*	49.49±14.01	57.25±16.01	0.076
*Bacteroidaceae*	33.7±17.18	43.84±18.34	0.080
*Rikenellaceae*	6.21±7.25	7.23±5.33	0.615
*Prevotellaceae*	6.48±13.97	1.33±3.52	0.417
*Porphyromonadaceae*	1.76±1.34	2.99±2.59	0.106
*Barnesiellaceae*	0.37±0.35	0.86±0.65	**0.001**
*Odoribacteraceae*	0.44±0.26	0.66±0.40	**0.009**
*Paraprevotellaceae*	0.42±0.58	0.26±0.66	0.417
*S24-7*	0.31±0.65	0.06±0.11	0.124
*FIRMICUTES*	49.12±13.88	40.41±15.87	0.085
*Ruminococcaceae*	25.29±7.73	20.37±9.17	0.044
*Lachnospiraceae*	13.08±4.20	12.48±7.16	0.753
*Clostridiaceae*	1.65±1.63	1.00±1.17	0.181
*Erysipelotrichaceae*	0.33±0.33	0.32±0.22	0.929
*Christensenellaceae*	0.22±0.41	0.39±0.55	0.284
*Turicibacteraceae*	0.01±0.02	0.02±0.02	0.043
*Veillonellaceae*	0.30±0.54	0.25±0.32	0.728
*PROTEOBACTERIA*	0.63±0.56	1.25±2.34	0.282
*Alcaligenaceae*	0.27±0.26	0.38±0.35	0.110
*Enterobacteriaceae*	0.07±0.2	0.01±0.01	0.144
*Desulfovibrionaceae*	0.01±0.01	0.02±0.03	0.765
*TENERICUTES*	0.26±0.29	0.39±0.61	0.384
*Anaeroplasmataceae*	0.03±0.01	0.03±0.01	0.992
*EURYARCHAEOTA*	0.03±0.064	0.04±0.09	0.639
*Methanobacteriaceae*	0.05±0.01	0.1±0.3	0.514
*VERRUCOMICROBIA*	0,15±0.29	0.08±0.12	0.384
*Verrucomicrobiaceae*	0.10±0.28	0.04±0.09	0.357
*Cerasicoccaceae*	0.03±0.09	0.03±0.09	0.998
*LENTISPHAERAE*	0.02±0.03	0.01±0.25	0.173
*Victivallaceae*	0.01±0.04	0.1±0.25	0.173
*ACTINOBACTERIA*	0.063±0.04	0.083±0.09	0.329
*Coriobacteriaceae*	0.035±0.04	0.028±0.06	0.695
*Bifidobacteriaceae*	0.01±0.02	0.05±0.05	0.290
*FUSOBACTERIA*	0.001±0.006	0.025±0.10	0.338
*Fusobacteriaceae*	0.00±0.00	0.025±0.1	0.338

Phyla and families in which the percentage of any of the 2 groups was >0.01% are shown. Data represent the mean±standard deviation. Differences were considered significant (in bold) at p<0.01 (adjusted p-value corrected for false discovery rate).

### Enterotype classification

**Fig 4:**
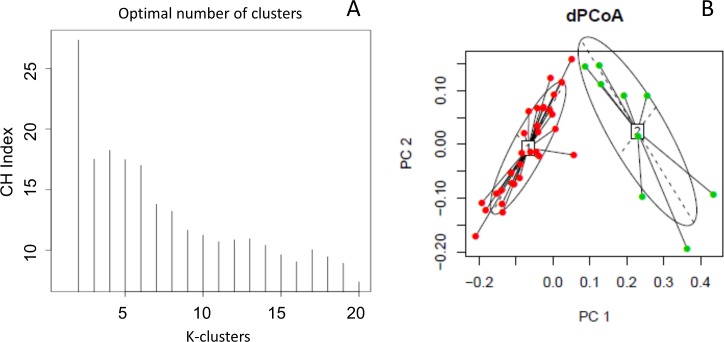
**Enterotype classification** A) k refers to clustering using the Calinski-Harabasz index B) PcoA plots using the Jensen-Shannon distance.

PAM analysis favored partitioning into two clusters (Fig 4A), the *Bacteroides* and *Prevotella* enterotypes, whereas the *Ruminococcus* enterotype was not present in our samples (Fig 4B). Seventy-seven percent of the samples were within the *Bacteroides* enterotype and the remaining 23% belonged to the *Prevotella* enterotype, in which 66% of the population was from the active group.

### Correlation of gut microbial taxa, body composition and physical activity parameters

**Fig 5:**
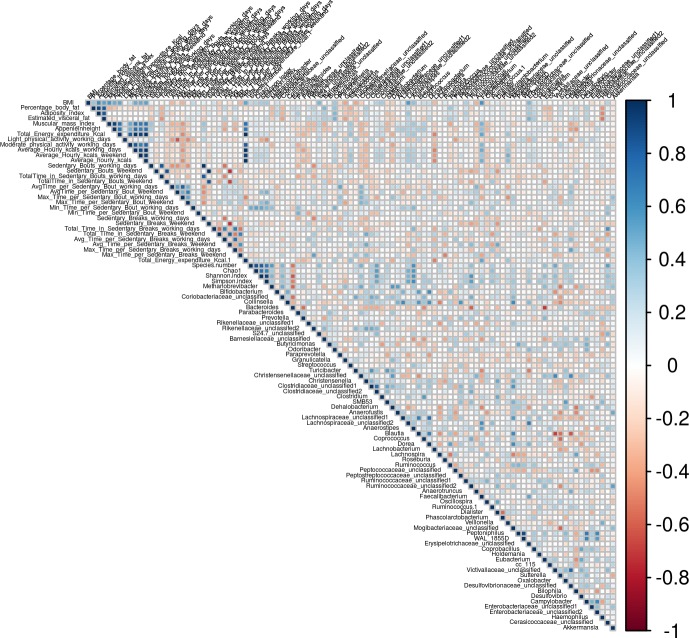
Spearman correlation of microbiota genera, body composition and physical activity parameters.

Bivariate correlation analysis was performed to study the associations between different variables and their influence on the microbiota. Body composition parameters did not correlate significantly with the percentage of *Firmicutes* or *Bacteroidetes* phyla and the *Firmicutes*/*Bacteroidetes* (F/B) ratio. At the family level no correlations were found. At the genus level, *Odoribacter* was positively associated with BFP, AI and VAT (ρ = 0.43, p = 0.001; ρ = 0.44, p = 0.008; ρ = 0.40; p = 0.018, respectively), whereas *Turicibacter* was negatively associated with BMI (ρ = -0.41, p = 0.016) and *Haemophilus* was negatively correlated with BFP, AI and VAT (ρ = -0.40, p = 0.02; ρ = -0.43, p = 0.01; ρ = -0.51, p = 0.002, respectively) (Fig 5). Regarding genera associated with muscular parameters, *Faecalibacterium* was positively associated with MMI (ρ = 0.421, p = 0.013) and AppMI (ρ = 0.416, p = 0.008) and *Coprococcus* and *Lachnospiraceae* unclassified1 were positively correlated with AppMMI (ρ = 0.40, p = 0.002; ρ = 0.43, p = 0.001, respectively) (Fig 5). Interestingly, the minimum time in sedentary bouts was significantly associated with the number of species and Shannon and Simpson indices (Fig 5), whereas light physical activity was negatively associated with both indices (Fig 5). Among other genera, *Odoribacter* was positively associated with the minimum time per sedentary bout, whereas *Paraprevotella* correlated with the average time per sedentary bout (ρ = 0.52, p = 0.002), *Desulfovibrionaceae* unclassified correlated with the maximal time per sedentary break (ρ = 0.53, p = 0.004), and *Akkermansia* with the maximal time per sedentary bout (ρ = 0.42, p = 0.03) (Fig 5).

### Multiple regression analysis

Multiple regression analysis was performed to establish the influence of the different variables to the differences observed at genera level between the active and sedentary groups. None of the variables considered for physical activity and body composition had an impact on *Turicibacter*, *Bifidobacterium*, *Odoribacter*, *Ruminococcus* and *Ruminococcus* unclassified2 genera, whereas the unclassified genus of the *Barnesiellaceae* family and *Haemophilus* were predicted by the percentage of body fat, and *Coprococcus* and the *Ruminococcae* family unclassified1 genus were partially explained by muscular mass parameters. Interestingly, the unclassified genus of the *Desulfovibrionaceae* family and the *Paraprevotella* genus were predicted by sedentary behavior (Table 4).

**Table 4:** Multivariate linear regression analysis of the bacterial groups significantly different between active and sedentary women as dependent variants, and diet composition, fecal water, body composition parameters and physical activity parameters as independent variables.

***Turicibacter***	**B**	**p**	**R^2^**
Dairy products	0.503	<0.001	0.424
Cereals	-0.377	0.007	
***Haemophilus***	**B**	**p**	**R^2^**
Fecal water (%)	0.444	0.007	0.512
Body Fat (%)	-0.555	0.001	
***Desulfovibrionaceae unclassified***	**B**	**p**	**R^2^**
Maximum Time per sedentary bout	0.578	0.002	0.334
***Bifidobacterium***	**B**	**p**	**R^2^**
Protein (%)	-0.484	0.002	0.234
***Paraprevotella***	**B**	**p**	**R^2^**
Average time per sedentary bout	0.438	0.025	0.192
***Odoribacter***	**B**	**p**	**R^2^**
Fiber	-0.344	0.032	0.225
Diet lipids (%)	0.331	0.038	
***Ruminococcus***	**B**	**p**	**R^2^**
None			
***Ruminococcaceae* unclassified1**	**B**	**p**	**R^2^**
Diet lipids (%)	-0.426	0.009	0.201
***Ruminococcaceae* unclassified2**	**B**	**p**	**R^2^**
Diet lipids (%)	-0.459	0.004	0.211
***Barnesiellaceae* unclassified**	**B**	**p**	**R^2^**
Body fat (%)	0.386	0.029	0.149
***Coprococcus***			
AppMMI (kg/m2)	0.389	0.023	0.151

AppMMI: appendicular muscular mass index.

### Fecal enzymatic activity

Bacterial enzymes in the intestine are involved in the metabolism of foods and drugs, and changes to the composition of intestinal bacterial populations may result in modifications to the bacterial enzyme profile that could impact host health status. Accordingly, enzymatic activities of fecal samples were compared between the active and sedentary groups. The activity of cysteine aminopeptidase in feces was significantly higher in the active group than in the sedentary group (6.6-fold higher, p = 0.033), whereas no differences were detected for other enzyme activities. In correlation analysis, cysteine aminopeptidase activity was negatively correlated with the presence of *Bacteroides* (ρ = -0.39, p = 0.013), whereas α-fucosidase activity was positively correlated with the presence of *Bifidobacterium* and *Odoribacter* genera (ρ = 0.52, p = 0.0005; ρ = 0.45, p = 0.003, respectively) and alkaline phosphatase was negatively correlated with *Desulfovibrio* genus (ρ = -0.42, p = 0.007).

## Discussion

Physical exercise is a low-cost strategy to prevent a wide variety of diseases. One area where physical exercise may have beneficial effects is the gut microbiota, an environmental factor whose imbalance or dysbiosis is associated with various types of diseases including inflammatory bowel disease (IBD), colon cancer, cardiovascular diseases and diabetes, among others. We sought to test whether exercise practiced at a dose recommended by WHO was sufficient to alter the composition of the gut microbiota in women. Our findings indicate that whereas physical exercise did not produce significant changes to the microbiota diversity/richness, sedentary parameters (i.e., sedentary time and breaks) correlated with microbiota richness. Our results agree with those of Welly et al., who also failed to find an increase in microbiota richness in an experimental model of exercise performed spontaneously by animals [39]. By contrast, an increase in microbiota diversity has been reported in professional rugby players [40], and also in animal models in which exercise was performed voluntarily, at high intensity, or forced [16,18,41]. This discrepancy in findings may reflect different factors, such as the modality of exercise (voluntary or forced), intensity, and time in life when exercise is performed [22,42]. In a study comparing voluntary versus forced exercise on gut microbiota, forced exercise was found to produce more marked effects on microbiota diversity than voluntary exercise [39]. A greater diversity of bacterial species has been related to better health conditions [25], and low diversity has been linked to obesity and several diseases such as diabetes [43], IBD [44], colon cancer [45], and autism [45]. Accordingly, it would be of great interest to establish the type and dose of exercise that would be sufficient to increase microbial diversity in the gut.

A decrease in the *Bacteroidetes*/*Firmicutes* ratio has been related to obesity, suggesting that the normalization of this ratio might prevent obesity-associated diseases [46]. The effect of exercise on *Bacteroidetes* and *Firmicutes* in animal models is still controversial. Evans et al. detected an increase in *Bacteroidetes* and a decrease in *Firmicutes* that was reflected in an increased B/F ratio [15], whereas Kang et al. [18] reported a decrease in *Bacteroidetes* and an increase in *Firmicutes*, reflecting a decrease in the B/F ratio. In the present study, no changes were detected in *Bacteroidetes* and *Firmicutes*; however, we detected a trend for a lower presence of the *Bacteroidetes* population in the active group, which is consistent with findings in professional athletes [40]. Based on the study by Arumugam et al. [32], we performed an enterotype classification of our samples and we found that only 23% of the population belonged to the *Prevotella* enterotype, with twice as many active than sedentary women within this enterotype. The *Prevotella* enterotype has been linked to long-term consumption of carbohydrate-rich diets, whereas the *Bacteroides* enterotype has been related to a diet rich in protein and animal fat [47]; however, we did not find any relationship between enterotypes and the carbohydrate or protein content of the participant’s diets. Recently, this enterotype classification scheme has been questioned as statistical analysis of a large number of samples of microbiota communities has shown that aside from these discrete clusters, continuous gradients (enterogradients) of the dominant genera can be found that could be better considered as biomarkers of the microbiota, and might more accurately associate with disease or health markers than a discrete enterotype cluster [48,49].

Among all the genera studied, the abundance of eleven of them was significantly different between the active and sedentary group, with *Paraprevotella* and an unclassified genus of the *Desulfovibrionaceae* family specifically associated with sedentarism parameters, while the remaining genera where largely associated with diet parameters. Diet is one of the known factors that most influences gut microbiota and to ascertain its influence in human studies is challenging [14]. Individuals who perform high levels of exercise often have special diets [40]. In our study, the physical exercise load was the minimum recommended by WHO for adults aged 18–64 years, which clearly does not require an extreme diet. Nonetheless, as exercise and diet often go hand in hand, an active lifestyle is frequently associated with a high consumption of fruits and vegetables, whereas sedentarism is associated with the consumption of high-calorie and fatty foods. Indeed, exercise interventions in human populations have resulted in an improvement in diet habits [50–52]. Although the diets were similar in our study regarding total carbohydrates, protein and fat content, significant differences were observed for fiber (higher in the active group) and processed meat (higher in the sedentary group). Moreover, we found an inverse correlation between fat intake and muscle parameters, and between fiber intake and body fat composition, which prevented multiple regression analysis of dietary factors and exercise-related factors together because of collinearity problems. According to studies conducted in rodents, females who performed physical exercise had a lower proportion of the *Turicibacteraceae* family and *Turicibacter* genus [17,42]. Cereal consumption has been shown to modify *Turicibacter* abundance in microbiota in animals [53], and therefore alterations in the *Turicibacter* genus and its relationship with exercise in humans deserves further research. Somewhat surprisingly, a marked increase in the *Haemophilus* genus, which includes some species related to pathogenicity, was detected in active women. Whether a sizeable presence of *Haemophilus* in gut microbiota can have a positive or negative effect on health remains unknown. An increase in the abundance of *Haemophilus* has been described in patients with multiple sclerosis [54], and has also been associated with colorectal carcinoma in patients with adenoma [55]. Conversely, a decrease in *Haemophilus* spp. has been linked to the development of type II diabetes [56], and a complete depletion of *Haemophilus* spp. was detected in patients with rheumatoid arthritis and negatively correlated with the level of serum autoantibodies [57].

The alterations in the levels of an unclassified genus of the *Desulfovibrionaceae* family and the *Paraprevotella* genus were partially explained by the disruption of sedentary lifestyle. To our knowledge, this is the first demonstration of a correlation between the presence of certain bacterial taxa and the disruption of sedentary lifestyle. The *Paraprevotella* genus is one of four comprising the *Prevotellaceae* family, and is characterized by the production of succinic and acetic acid as major fermentation products. Only two species have been described from this genus and its potential effects on human health are unknown [58]. In line with previous results, the *Coprococcus* genus was more abundant in active women [42]. A low presence of *Coprococcus* has been linked to IBD and a lower treatment response in pediatric patients [59], and also to decreased resistance to *Campylobacter* infection [60], and to an increased severity of atopic disease [61]. *Coprococcus* is a butyrate-producing genus, which could explain some exercise health-promoting effects [62,63]. An unclassified genus of *Barnesiellaceae* was also modified by exercise. The implication of these bacterial genera in the intestinal microbiota and their possible role in the human body await further investigation.

Species analysis by qPCR showed that the abundance of *R*. *hominis*, *A*. *muciniphila* and *F*. *prausnitzii* was significantly higher in active women than in their sedentary peers. These species have been related to health-promoting effects. A high abundance of *A*. *muciniphila* has been previously described in the microbiota of athletes [40], while low levels have been linked to metabolic disorders (obesity, metabolic syndrome and type II diabetes) in patients with IBD [64]. In animal studies, the presence of *A*. *muciniphila* has been associated with the existence of other beneficial bacteria such as *Bifidobacterium* [65]. Although a similar finding was found in our study, with the *Bifidobacterium* genus more abundant in the active group, an interventional study would be required to test if there is a co-increment of *Bifidobacterium* and *A*. *muciniphila* as the exercise regimen increases. Both *R*. *hominis* and *F*. *prausnitzii* have a beneficial effect on health as they produce butyrate, which has a positive impact on intestinal health and lipid metabolism [66,67]. *F*. *prausnitzii* also produces metabolites with anti-inflammatory action [68]. As described, some bacterial genera were associated with the break of sedentary behavior. This may be important for health because time in sedentary behavior has been associated with the increased risk of metabolic syndrome, type 2 diabetes and cardiovascular disease [69], whereas breaks in sedentary time have been linked to benefits in triglyceride and glucose levels, waist circumference and body mass index [70].

The determination of fecal enzymatic activities revealed a dramatic (6.6-fold) increase in cysteine aminopeptidase activity in active women versus sedentary women. Bacterial cysteine aminopeptidases releases N-terminal cysteine residues from polypeptides and proteins [71], which can serve as energy sources for bacteria that metabolize the protein or for other bacterial species [72]. While bacterial gut protein metabolism is proportionally less significant than carbohydrate metabolism, it is nontheless important because the reaction products produced, including short-chain fatty acids, ammonia, phenols and dicarboxylic acids, can influence host health [72]. Although little is known about specific enzymatic activities of microbiota bacterial groups, cysteine aminopeptidase activity is widely found in the *Lactobacillales* order [73]; however, we did not find significant differences at any level within this group of bacteria. Further research is needed to understand how changes in microbiota profiles provoke changes in the metabolic profile of the commensal microbiota.

In conclusion, the results presented here together with previous findings show that physical activity modulates the microbiota profile. The dose of exercise that has been considered in our study is the minimum that is recommended by WHO and can thus be easily implemented by individuals in their daily life. Our results indicate that physical activity performed at low doses but continuously can increase the abundance of health-promoting bacteria (*Bifidobacterium spp*, *R*. *hominis*, *A*. *muciniphila* and *F*. *prausnitzii)* in the microbiota. Although we failed to find an increase in microbiota richness, we found an inverse association between sedentary parameters and microbiota richness (number of species, and Shannon and Simpson indices), which might indicate that perhaps not only is the dose and mode of exercise important, but also the pattern of exercise, such as breaks in sedentary time, avoiding long periods of inactivity in daily life, to induce changes in gut microbiota.

## Supporting information

S1 Fig **Melting curve dissociation analysis of *Bifidobacterium longum* (A) and *Roseburia hominis* (B)**.
(TIF)

S2 Fig Observed species, Chao1, and phylogenetic distance whole tree rarefaction curves. Plotted red line is the mean±standard deviation of active women samples and blue line is the mean±standard deviation of sedentary women samples.
(TIF)

S1 Table Primers and amplification conditions for real-time PCR. (DOCX)
