# Supplementary figures and images for: Differences in gut microbiota profile between women with active lifestyle and sedentary women
Source: PLoS One. 2017 Feb 10;12(2):e0171352. doi: 10.1371/journal.pone.0171352 (PMC5302835; doi:10.1371/journal.pone.0171352)

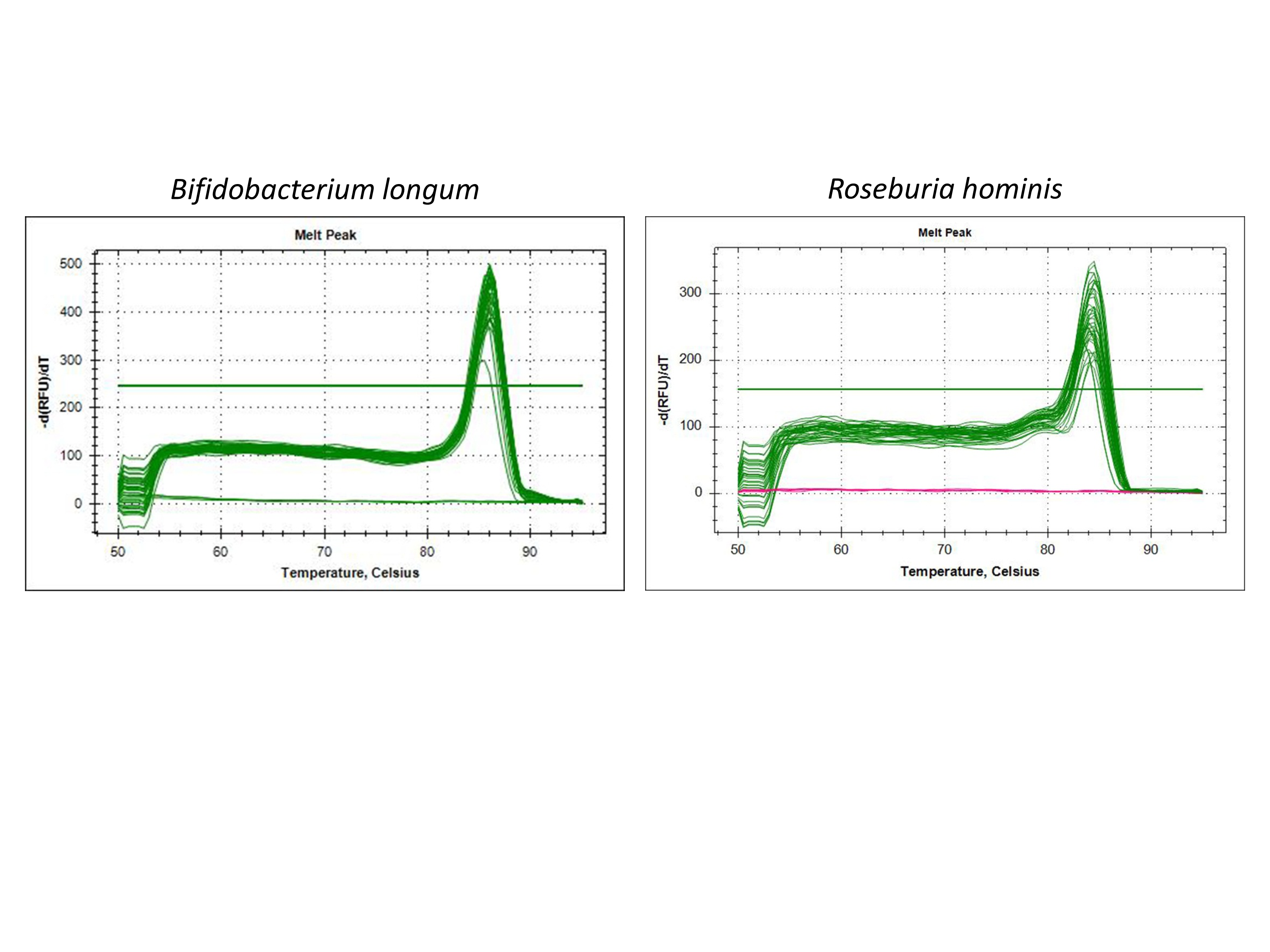

Supplement: S1 Fig — Melting curve dissociation analysis of Bifidobacterium longum (A) and Roseburia hominis (B). (TIF) [file pone.0171352.s001.tif]

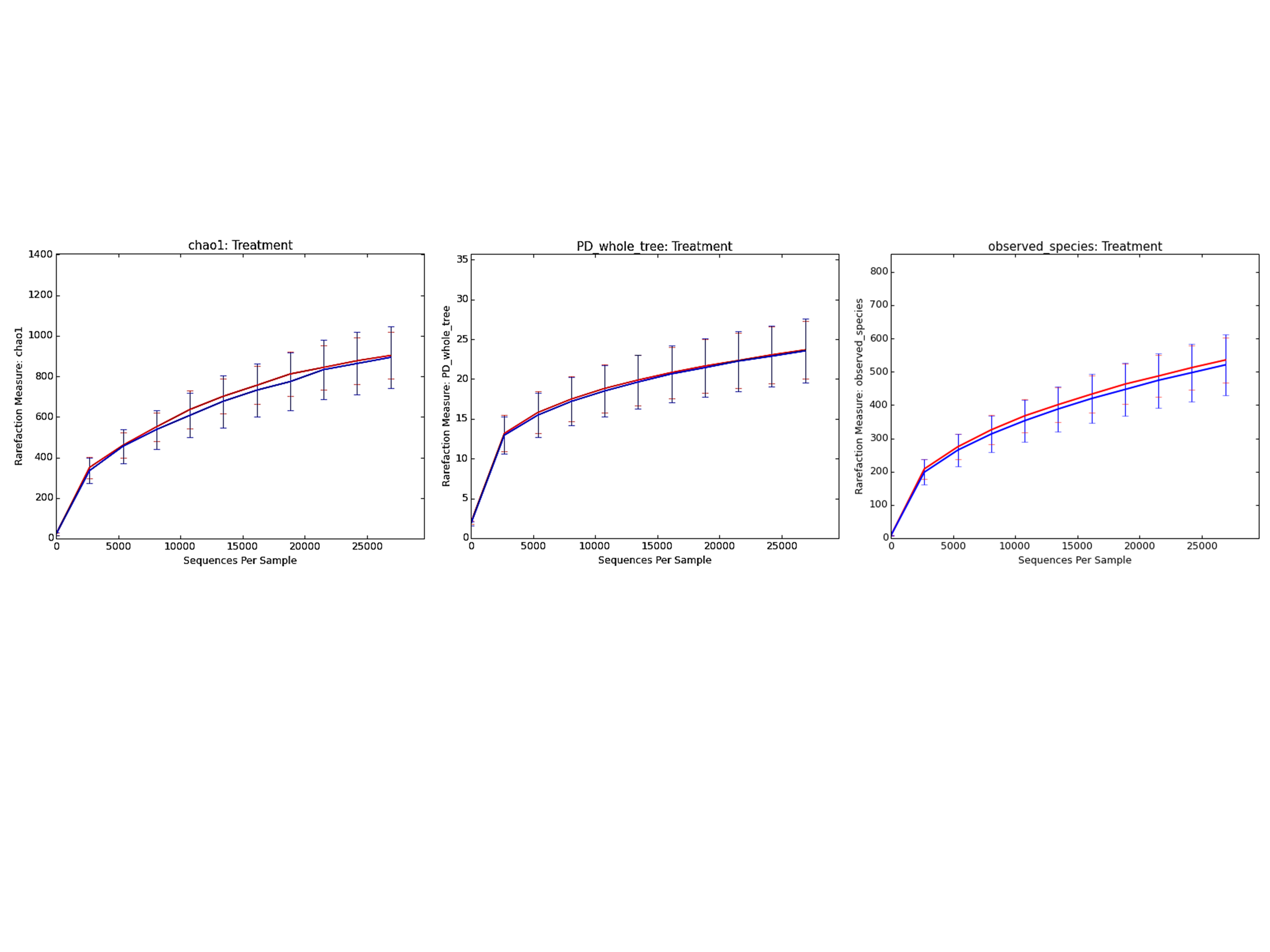

Supplement: S2 Fig — Plotted red line is the mean±standard deviation of active women samples and blue line is the mean±standard deviation of sedentary women samples. (TIF) [file pone.0171352.s002.tif]
