# Supplementary material for: Differences in gut microbiota profile between women with active lifestyle and sedentary women
Source: PLoS One. 2017 Feb 10;12(2):e0171352. doi: 10.1371/journal.pone.0171352 (PMC5302835; doi:10.1371/journal.pone.0171352)
Supplement: S1 Table — (DOCX) [file pone.0171352.s003.docx]

Supplemental Table 1: Primer characteristics and amplification conditions for real-time PCR

| Primers | Sequence 5’-3’ | Annealing temperature (ºC) | Specificity (%) | Efficiency (%) | Melting temperature (°C) | Reference |
| --- | --- | --- | --- | --- | --- | --- |
| Total Bacteria 16S (27F/518R) | F: 5’ –GAG TTT GAT CMT GGC TCA G-3’  R: 5’ –ATT ACC GCG GCT GCT GG-3’ | 58 |  | 91.3 | 84 | [1] |
| *Roseburia hominis.* | F: 5´-GCA CTT TAA TTG ATT TCT TCG G-3’  R: 5´-TCT TAG TCA GGT ACC GTC ATT C-3’ | 59 | 100 | 93.5 | 84.5 | This work |
| *Faecalibacterium prausnitzii* | F: 5´-CCC TTC AGT GCC GCA GT-3’  R: 5´-GTC GCA GGA TGT CAA GAC-3´ | 48 |  | 93.2 | 81 | [2] |
| *Bifidobacterium longum* | F: 5´-GGA TCC ATC AGG CTT TGC TTG G-3’ R: 5´-CGG GTA AAC TCA CTC TCG C-3’ | 57 | 100 | 94.7 | 86 | This work |
| *Akkermansia muciniphila* | F: 5´- CAG CAC GTG AAG GTG GGG AC-3’  R: 5´-CCT TGC GGT TGG CTT CAG AT-3’ | 49 |  | 95.1 | 87 | [3] |

**References**

1. Brankatschk R, Bodenhausen N, Zeyer J, Burgmann H. Simple absolute quantification method correcting for quantitative PCR efficiency variations for microbial community samples. Applied and Environmental Microbiology. 2012;78(12):4481-9. doi: 10.1128/AEM.07878-11 [doi].

2. Son JS, Zheng LJ, Rowehl LM, Tian X, Zhang Y, Zhu W, et al. Comparison of fecal microbiota in children with autism spectrum disorders and neurotypical siblings in the Simons Simplex Collection. PloS one. 2015;10(10):e0137725.

3. Collado MC, Derrien M, Isolauri E, de Vos WM, Salminen S. Intestinal integrity and Akkermansia muciniphila, a mucin-degrading member of the intestinal microbiota present in infants, adults, and the elderly. Applied and Environmental Microbiology. 2007;73(23):7767-70. doi: AEM.01477-07 [pii].
